# Psychometric properties of the American version of the Chronic Uncertainty scale: long and short version (CU-40; CU-20)

**DOI:** 10.1186/s40359-024-02206-6

**Published:** 2024-11-27

**Authors:** Shiwa Ghassabei, Walid A. Afifi, Tamara Afifi, Katja Petrowski

**Affiliations:** 1 Medical Psychology & Medical Sociology, University Medical Center of the Johannes Gutenberg University of Mainz, Duesburgweg 6, 5th, 05317, Mainz, 55128 Germany; 2 Department of Communication, University of California-Santa Barbara, Santa-Barbara, USA

**Keywords:** Chronic uncertainty, Exploratory factor analysis, Confirmatory factor analysis, Psychometric properties

## Abstract

**Background:**

Uncertainty is a widespread phenomenon experienced worldwide. The bulk of existing research to date has focused on transitory or acute experiences of uncertainty, often in the particular context of illness. The current study evaluated the psychometric properties of the cross-contextual 40-item (long) and 20-item (short) Chronic Uncertainty scale (CU-40; CU-20).

**Methods:**

A U.S. sample of 1083 participants (55% female) was recruited via Mechanical Turk (MTurk). Exploratory factor analysis revealed a six-factor model.

**Results:**

Results of the Confirmatory factor analysis showed good model fit for the original and a slightly better model fit for the modified version of the CU-40 and CU-20. Slightly different item-to-factor attributions were suggested for the questionnaires. Internal consistencies were good for both models.

**Conclusion:**

The evidence suggests that the CU scale offers psychometrically sound assessments of chronic uncertainty across a range of dimensions. Further assessments and implementations of the CU in different contexts using diverse samples are encouraged to test the efficacy of the CU measures as screening tools of general aspects of chronic uncertainty.

**Supplementary Information:**

The online version contains supplementary material available at 10.1186/s40359-024-02206-6.

## Introduction

In recent years, there have been many simultaneous crises or disasters across the globe (e.g., pandemic, climate crisis, war, displacement) that have caused uncertainties across large segments of the world’s population. Broadly, uncertainty is defined as “a dynamic state in which there is a perception of being unable to assign probabilities for outcomes” (Penrod, 2001, p. 241). Uncertainties are multiplicative and simultaneous; not only does uncertainty about one issue often create uncertainties about others, but individuals typically manage multiple related uncertainties at the same time. In addition, part of the challenge of management is that outcomes of an issue over which one has uncertainty can be either knowable (e.g., uncertainty about the outcomes of a genetic test) or unknowable (e.g., when the next pandemics will occur [1]). Capturing the range of approaches to the study of this construct, Afifi and Afifi [2], in their discussion of the Covid-19 pandemic as an “uncertainty shock,” identified uncertainty as “an inability to predict outcomes [of] a typically important issue” (p*.*326) and noted that it can be experienced as both acute (i.e., transitory) and chronic (i.e., persists for months or years). This investigation examines the soundness of the Chronic Uncertainty Scale [2, 3] as an assessment of individuals ongoing experiences of uncertainties.

### Impacts of uncertainty

Threat-related uncertainty has been associated with a wide range of negative outcomes. The most commonly studied outcome is stress [4–6], but others include mental health deficits [7], depression [8], hopelessness [9], avoidance behavior (e.g., information-seeking avoidance [10], travel avoidance [11], and disease [12], among other negative states. A chronic state of uncertainty, while under-studied, is especially problematic because it is likely to produce allostatic load [12]—the “overactivity of the stress hormone axis” ([13], p.110) and breakdown of the healthy stress response system—along with the wide-ranging consequences that often result [14].

Despite growing awareness of the critical nature of ongoing experiences of uncertainty, measures of chronic uncertainty that capture their multi-dimensional and potentially simultaneous effects have been lacking. Existing instruments are either focused on acute states of uncertainty [15], are context-bound (e.g., illness-related uncertainty; Mishel’s Uncertainty in Illness Scale [16]) or capture individual differences in uncertainty (e.g., Intolerance of Uncertainty [17]). This latter group of measures has received the most attention from scholars, especially as it relates to responses to uncertainty events/shocks. Two of the most popular dispositional approaches to uncertainty are Intolerance of Uncertainty (IUS) [17] and Need for Closure [18].

*Intolerance of Uncertainty* represents “an individual's tendency to interpret ambiguous situations as threatening and to respond to novel, complex and insoluble situations with discomfort and avoidance” ([19], p.1199). It has been shown to be strongly linked to both anxiety and worry [20]. *The Need for Closure* is a trait characterized by the need for predictability and structure. Specifically, it reflects a preference for "an answer on a given topic, any answer, … compared to confusion and ambiguity" ([21], p.337) and is associated with the tendency of individuals to “seize” on information that addresses their uncertainty and quickly “freeze” on that explanation [22]. It has also been shown to be activated under conditions of threat or loss [23] and linked to heightened attraction to extremist ideologies [24].

With an eye toward understanding uncertainty as a consequence of structural conditions that create chronic states of existence, Afifi and colleagues developed the *Chronic Uncertainty* (CU) scale, believed to be the first self-report measure of chronic uncertainty that is intended to capture the multi-dimensional and simultaneous experience of multiple chronic uncertainties [2]. The CU emerged from the study of populations in three different chronic uncertainty situations: (1) Palestinian refugee camps in Lebanon [25], (2) undocumented immigrants in the United States [25], and (3) survivors of a Category-Five hurricane in the United States [26]. The experience of those communities, together with a thorough review of literature, led Afifi and Afifi [2] to propose six common types of chronic uncertainty: (1) personal safety (Safety), (2) security of the community in which they live (Country), (3) financial comfort (Finances), (4) personal health and wellbeing (Health), (5) the future of their close relationships (Relational), and (6) the wellbeing of close others (Other wellbeing). The authors also explicitly noted that the experience of uncertainty is context-specific and that other types of chronic uncertainty may emerge for particular communities (e.g., the threat of deportation/separation from family).

Unlike dispositional measures such as the *Intolerance of Uncertainty scale* [17], the CU scale is designed to capture the extent to which individuals experience long-term, structural-caused uncertainties in their environment. Higher scores on the CU scale reflect a state where individuals face prolonged conditions that often limit their ability to predict significant life outcomes, rather than indicating personal preferences or situation-specific variance. The measure is intended to assess the uncertainties that emanate from typically enduring structural factors, such as displacement, poverty, or prolonged conflict, on individuals and communities.

Although this investigation represents the first empirical examination of the CU, as originally developed (in English), a prior study investigating the psychometric properties of the 20-item version of the German-translated CU revealed good factor structure and construct validity [3]. The aim of this study is to investigate the psychometric properties of the full- and brief-version of the English-language CU using a large sample gathered via Amazon Mechanical Turk Prime (MTurk) in the United States (U.S.).

## Method

One-thousand and eighty-three participants (*N* = 1083) were recruited through an Amazon Mechanical Turk (MTurk) Prime account. Eligibility parameters included that they be at least 18 years old and that they reside in the United States. The data were collected in November, 2017. The typical payment for MTurk workers at the time was $2/hour (Hara et al., 2018). Participants in this study were paid $2 for a 30-min survey (i.e., twice the typical hourly rate).

Several attention checks were included in the survey. Participants had to pass several tests of the attention checks. First, people missed two or more attention checks were excluded from analysis (*n* = 14). In addition, those who missed either the third attention check ("Please click the bubble under the neutral column."; *n* = 17)) or the 5th ("If you are paying attention, please click "strongly disagree."; *n* = 33) were also excluded. Finally, participants who completed less than 75% of the questions of interest for this investigation and those who completed the survey in two standard deviations less than the average completion time (i.e., 12 min or less), were excluded, resulting in a final sample size of 773.

The majority of the remaining 773 participants identified as female (55%, *n* = 426) and white (79%, *n* = 586; followed by Black (10%, *n* = 77), Asian (6%, *n* = 46), and Latin (3%, *n* = 20)). The highest education most commonly achieved by participants was college graduate (42%, *n* = 327), followed by partially completed college (35%, *n* = 267); the majority of the sample reported income less than $50,000 (51%, *n* = 397; followed by $50,000—$99,999 (37%, *n* = 288), and $100,000—$149,999 ((9%, *n* = 68), and the average age was 38.36 (*SD* = 29.34). Forty-four percent (*n* = 340) were never married, 43% (*n* = 335) were married, and 10% (*n* = 77) indicated being divorced (with the remaining participants indicating being widowed or separated). Finally, of the 51% (*n* = 382) who reported having children, the average number of children was 2.11 (*SD* = 1.14).

### Chronic uncertainty scale

The Chronic uncertainty scale is comprised of 40 items in the long version (CU-40) and 20 items in the short version (CU-20) [27]. The full version includes six domains of uncertainty: (1) *Safety and security*, (2) *Country’s safety and security*, (3) *Finances*, (4) *Health*, (5) *Other’s well-being*, and (6) *Relationship security*, each with 4–6 items. Because the threat of deportation and family separation was salient in the U.S. in 2017, and to acknowledge the flexibility of the CU as a measure that can incorporate community-specific uncertainties, two items were added to assess chronic uncertainty about the potential for family separation resulting in a seventh factor: (7) *Threat of separation from family*.

The instructions for the scale were as follows:

*The following questions ask about your level of chronic uncertainty about several aspects of your life. Uncertainty is a belief that you’re not able to predict the outcome of an issue. When something is chronic, it means that it is ongoing. In each case, you will be asked to think about the average level of uncertainty you have experienced during the past year.*

Participants were then asked to rate each item according to “How uncertain have you been in the past year about…” (bolded), using a 6-point Likert-type scale ranging from 1 (“Extremely Certain”) to 6 (“Extremely Uncertain”).

### Statistical analyses

Analyses were conducted using R v1.4.1106 (R Core Team, 2018) and associated packages of psych [28] and lavaan [29] using the cfa, semPath, and semTools functions. Additionally, the package of MBESS was used [30].

Two separate Principal axis factor analysis, a type of exploratory factor analysis (EFA) was conducted to examine if the factor structures of the CU-20 and CU-40 are equivalent. To evaluate if the present data fit the EFA, the Kaiser-Mayer-Olkin Criterion (KMO) was applied. KMO-values between 0.80 and 1 indicate good sample adequacy, between 0.70 and 0.79 sample adequacy is moderate, between 0.60 and 0.69 sample adequacy is mediocre. KMO-values below 0.06 refer to sample inadequacy [31]. Additionally, scree-plots, incremental variance were examined for factor retention. To test sphericity, the bartlett’s test was conducted. A significant bartlett’s test indicates that the data are suitable for factor analysis [32].

Principle axis analysis was used because an underlying theoretical structure was assumed. The oblimin rotation was applied to consider possible intercorrelations of the extracted factors. For factor extraction, factors with an eigenvalue of 1.0 or higher were kept, which were graphically examined using a scree-plot [31, 32]. Factor loadings were examined to assign items to the extracted factors. Each item is assigned to the factor on which it loads the highest (see factor matrix; Tables 3 and 7). Items with factor loadings > 0.30 are suggested to represent the factor [33].

Two separate confirmatory factor analysis (CFA) were calculated for CU-40 and CU-20 respectively. To test assumptions of the CFA, normality was assessed with the Shapiro–Wilk test. The assumption of normality was violated. To correct for normality, the Satorra-Bentler correction was applied [34]. No further corrections were necessary.

CFAs were calculated using the maximum-likelihood estimation (ML). To evaluate model fit, the standardized root mean square residual (SRMR) and the root mean square error of approximation (RMSEA) as absolute fit index and the comparative fit index (CFI) and tucker-lewis index (TLI) as incremental fit index were used. Regarding CFI and TLI, values > 0.95 indicate a good model fit. Values for SRMR < 0.05 imply a better model fit, while values for RMSEA < 0.06 indicate good model fit [35]. Due to the relatively large sample size, adjusted chi-square (*x*^2^) was not used to evaluate goodness of fit. Furthermore, a multigroup confirmatory factor analysis (MGCFA) was conducted to determine measurement invariance across gender (group 1 = men; group 2 = women) and age (group 1: 18–29 years; group 2: 30–39 years; group 3: 40–49 years; group 4: 50–59 years; group 5: 60–69 years; group 6: 9 years; group 6: ≥ 70 years). MGCFA was performed using four hierarchical models: 1) configural, 2) metric, 3) scalar, and 4) strict model [36, 37]. Changes (∆) in goodness of fit indices (∆CFI, ∆TLI, ∆SRMR, ∆RMSEA) were calculated to assess measurement invariance. According to recommendations of Chen [36] and Milfont and Fischer [37], change of ∆CFI ≤ 0.01, ∆TLI ≤ 0.01, ∆SRMR ≤ 0.03, and ∆RMSEA ≤ 0.015 indicates measurement invariance.

McDonalds omega [38] was used to evaluate model reliability as it is considered a more accurate measure of internal consistency than Cronbach’s alpha [39]. Confidence intervals were set at 99% and 10,000-repetition bootstrap method were calculated. Higher values of omega infer good internal consistency [40].

## Results

### Descriptive statistics

Descriptive item statistics are provided in Table 1 (CU-40) and Table 2 (CU-20). Shapiro–Wilk tests for all items were significant (*p* < 0.001). To account for non-normality of the data, the Satorra-Bentler correction was applied.

**Table 1:** Descriptive statistics of the items – EFA-proposed CU-40

	Item	Mean	SD	Skew	Kurtosis	Shapiro–Wilk	Stand. Estimate
Safety	2	2.06	1.14	1.38	1.86	< .001	.70
	6	2.27	1.17	1.12	1.02	< .001	.78
	8	2.20	1.17	1.11	1.01	< .001	.65
	9	2.33	1.10	1.03	1.01	< .001	.71
	12	2.52	1.29	.87	.20	< .001	.62
	15	2.03	1.17	1.27	1.31	< .001	.55
	17	2.16	1.16	1.27	1.58	< .001	.78
	18	2.14	1.15	1.30	1.70	< .001	.84
	22	2.06	1.14	1.38	1.86	< .001	.87
	25	2.02	1.09	1.40	2.10	< .001	.86
	26	2.07	1.25	1.27	1.01	< .001	.67
	31	1.94	1.17	1.35	1.39	< .001	.71
	36	2.07	1.12	1.26	1.50	< .001	.89
	38	2.09	1.11	1.22	1.39	< .001	.88
	39	1.79	1.06	1.60	2.42	< .001	.60
	40	1.77	1.10	1.78	3.01	< .001	.63
Finances	7	3.08	1.52	.40	-.84	< .001	.85
	16	2.50	1.46	.88	-.11	< .001	.81
	21	2.76	1.52	.61	-.63	< .001	.89
	27	2.50	1.45	.85	-.17	< .001	.89
	29	2.91	1.53	.55	-.74	< .001	.70
	32	2.61	1.35	.76	-.11	< .001	.65
	35	2.91	1.57	.52	-.81	< .001	.89
Relationship security	3	2.38	1.39	1.03	.34	< .001	.91
	13	2.34	1.42	1.05	.28	< .001	.88
	19	2.42	1.46	.94	-.08	< .001	.92
	24	2.39	1.43	.98	.14	< .001	.93
	33	2.39	1.49	.98	-.04	< .001	.92
Country’s safety and security	4	3.65	1.44	-.02	-.91	< .001	.82
	11	3.63	1.40	.00	-.83	< .001	.80
	28	3.61	1.56	-.01	-1.08	< .001	.82
	37	3.39	1.49	.14	-.95	< .001	.70
Physical Health	1	2.26	1.25	.98	.25	< .001	.67
	10	2.60	1.34	.79	-.24	< .001	.72
	14	2.41	1.34	.96	.27	< .001	.85
	20	2.63	1.34	.76	-.18	< .001	.59
Mental Health	5	2.90	1.27	.53	-.29	< .001	.62
	23	2.90	1.61	.50	-.94	< .001	.82
	30	2.67	1.49	.70	-.50	< .001	.89
	34	2.79	1.52	.65	-.62	< .001	.91

*CU-40* Chronic Uncertainty Scale-long version

**Table 2:** Descriptive statistics of the items – EFA-proposed CU-20

	Item	Mean	SD	Skew	Kurtosis	Shapiro–Wilk	Stand. Estimate
Safety	22	2.06	1.14	1.38	1.86	< .001	.87
	25	2.02	1.09	1.40	2.10	< .001	.89
	36	2.07	1.12	1.26	1.50	< .001	.93
	38	2.09	1.11	1.22	1.39	< .001	.90
Finances	7	3.08	1.52	.40	-.84	< .001	.85
	21	2.76	1.52	.61	-.63	< .001	.89
	27	2.50	1.45	.85	-.17	< .001	.87
	35	2.91	1.57	.52	-.81	< .001	.90
Relationship security	19	2.42	1.46	.94	-.08	< .001	.93
	24	2.39	1.43	.98	.14	< .001	.93
	33	2.39	1.49	.98	-.04	< .001	.91
Country’s safety and security	4	3.65	1.44	-.02	-.91	< .001	.84
	11	3.63	1.40	.00	-.83	< .001	.80
	28	3.61	1.56	-.01	-1.08	< .001	.81
Health	1	2.26	1.25	.98	.25	< .001	.54
	14	2.41	1.34	.96	.27	< .001	.61
	30	2.41	1.34	.96	.27	< .001	.89
	34	2.67	1.49	.70	-.50	< .001	.90
Separation from Family	39	1.79	1.06	1.60	2.43	< .001	.81
	40	1.77	1.10	1.78	3.01	< .001	.86

*CU-20* Chronic Uncertainty Scale-short version

### Factor analysis

#### Long version of the CU

**Fig. 1:**
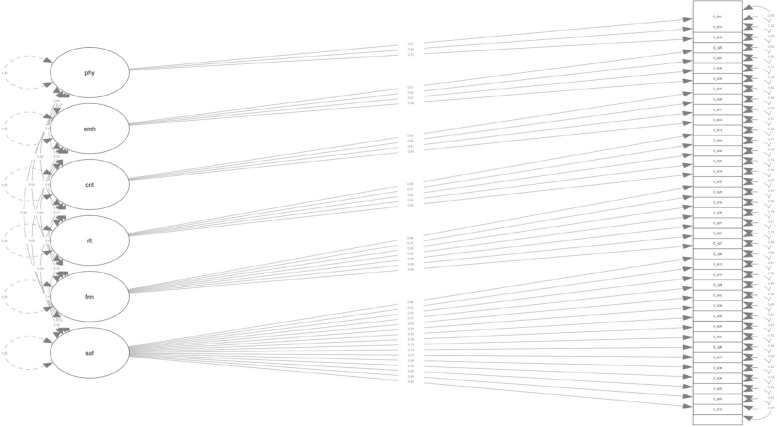
Factor structure of the adjusted Chronic uncertainty scale (CU-40) with standardized estimates. Notes. phy = physical health. emh = mental health. cnt = country’s safety and security. rlt = relationship security. fnn = finances. saf = safety

For the CU-40, the overall KMO value was > 0.96 and no item was lower than 0.87, indicating sample adequacy. Bartlett’s test of sphericity was significant (*X*^2^(39) = 895.23, *p* < 0.001). Parallel analysis scree-plot and eigenvalue inspection revealed a six-factor structure. Items were assigned to factors according to the factor loading matrix (Table 3, Fig. 1). Factor estimates ranged between 0.55 and 0.93 (Table 1). Descriptive statistics and the item to factor distribution of the adjusted CU-40 are provided in Table 1.

**Table 3:** Exploratory factor analysis: factor loading matrix – CU-40

Long version CU	Six-factor structure								
Item	Factor 1	Factor 2	Factor 3	Factor 4	Factor 5	Factor 6	h^2^	u^2^	com
1	-.02	.12	.03	.03	.53	.11	.44	.56	1.2
2	.53	.12	-.01	.02	.00	.18	.51	.49	1.3
3	-.03	.04	.92	.01	-.02	-.02	.84	.16	1.0
4	-.05	-.04	.00	.90	-.06	.03	.74	.26	1.0
5	.18	.04	.10	.13	.09	.33	.43	.57	2.4
6	.73	.07	-.07	.04	-.06	.15	.65	.35	1.1
7	-.08	.81	.04	.06	.02	.05	.73	.27	1.0
8	.36	.32	.08	.00	.08	.10	.55	.45	2.4
9	.50	.21	.03	.03	.02	.15	.58	.42	1.6
10	-.05	.00	-.05	-.02	.80	.05	.61	.39	1.0
11	.03	.06	-.02	.78	.04	-.06	.66	.34	1.0
12	.49	.01	.03	.03	.05	.20	.44	.56	1.4
13	.08	.01	.85	.00	-.01	-.02	.78	.22	1.0
14	.13	.00	..06	.02	.74	.05	.74	.26	1.1
15	.48	.03	.04	.02	.19	-.12	.34	.66	1.5
16	.03	.83	-.03	.00	-.02	-.04	..65	.35	1.0
17	.74	.06	-.06	-.01	.00	.11	.63	.37	1.1
18	.88	-.02	-.03	.02	.03	-.05	.73	.27	1.0
19	.02	-.01	.90	.00	-.01	.04	.84	.16	1.0
20	.17	.06	.08	.05	.29	.18	.38	.62	2.8
21	.02	.87	.00	.02	.06	-.05	.79	.21	1.0
22	.89	.00	-.01	.01	.05	-.07	.77	.23	1.0
23	.09	.07	.04	.10	.07	.61	.65	.35	1.2
24	-.03	-.04	.94	.02	.02	.04	.88	.12	1.0
25	.83	-.01	.08	.00	.07	-.07	.75	.25	1.0
26	.61	.03	.00	-.06	-.09	.26	.50	.50	1.4
27	.07	.91	-.01	-.06	-.06	.02	.82	.18	1.0
28	.03	.03	.05	.77	-.02	.06	.70	.30	1.0
29	.02	.60	.06	.07	-.01	.05	.50	.50	1.1
30	.03	.07	.09	.03	.15	.68	.78	.22	1.2
31	.65	-.01	.08	-.06	-.11	.22	.55	.45	1.3
32	.18	.43	.03	.07	.11	.07	.48	.52	1.6
33	.00	.02	.93	-.02	.01	-.02	.85	.15	1.0
34	.00	.06	.11	.10	.12	.70	.80	.20	1.2
35	-.07	.88	.03	.02	.03	.02	.80	.20	1.0
36	.85	.02	.06	.06	.01	-.06	.78	.22	1.0
37	.09	.00	-.03	.65	.08	-.02	.49	.51	1.1
38	.85	-.04	.02	.07	.05	-.03	.75	.25	1.0
39	.56	.08	.10	-.02	-.01	-.05	.39	.61	1.1
40	.57	.04	.16	-.05	.02	.00	.44	.56	1.2

*CU-40* Chronic Uncertainty Scale-long version. Principal axis extraction, oblimin rotation, h^2^ = communalities of all factors, u^2^ = uniqueness of items, com = Item complexity

Model fit of the EFA proposed six-factor model revealed a CFI of 0.91, TLI of 0.91, SRMR of 0.07, and RMSEA of 0.07 (Table 4). Modification indices inspection led to several adjustments. Item 20 (“The people closest to you getting through the day without physical pain”) was removed from the analysis because it loaded very low on each factor (0.05-0.29; see Table 2). Item 8 (“Your survival”) and item 9 (“The safety and security of the people closest to you”) loaded very high on the other subscales. Because they were assigned to the same factor, both items were included as residual covariance. The same logic applied to item 18 (“Avoiding being the victim of crime in your neighborhood”) and item 22 (“Avoiding harm from violence in your neighborhood”).

**Table 4:** Model fit indices for the long version of the CU-40

	CFI	TLI	SRMR	RMSEA
Theoretical 7-factor model	.88	.87	.06	.07
EFA-proposed 6-factor model	.91	.91	.07	.07
Adjusted 6-factor model	.92	.91	.06	.06

*CU-40* Chronic Uncertainty Scale-long version. Robust values

Item 4 and item 28 were considered as residual covariance, which led to decreased cross correlation with the other factors and slight improvements of the model fit. Additionally, item 22 (“Avoiding harm from violence in your neighborhood”) and item 36 (“Feeling secure in your neighborhood”) from the factor *Safety* correlated also highly with the other factors. After analyzing them as residual covariance, they correlated less with the other factors and led to slight improvements in model fit indices.

These adjustments improved the overall model fit: CFI = 0.92, TLI = 0.91, SRMR = 0.06, and RMSEA = 0.06 (Table 4). Model fit of the theoretically established seven-factor model showed a CFI of 0.88, TLI of 0.87, SRMR of 0.06, and RMSEA of 0.07 (Table 4). These model fits are not better than the adjusted 6-factor model, hence the adjusted 6-factor model was kept.

Measurement invariance analysis revealed metric, scalar and strict invariance across gender: ∆CFI ≤ 0.01, ∆TLI ≤ 0.01, ∆SRMR ≤ 0.03, and ∆RMSEA ≤ 0.015 (Table 5). The long version of the CU questionnaire is metric, scalar and strict invariant regarding ∆CFI and ∆TLI (≤ 0.01). Values for ∆SRMR are metric, scalar and strict invariant (≤ 0.03), as well as for ∆RMSEA ≤ 0.015 (Table 6) across age.

**Table 5:** Measurement invariance across gender

Model	N	CFI	∆CFI	TLI	∆TLI	SRMR	∆SRMR	RMSEA	∆RMSEA
Male	344								
Female	426								
Adjusted CU-40									
Configural		.916		.908		.064		.064	
Metric		.915	-.001	.91	.002	.074	.01	.064	0
Scalar		.908	-.007	.904	-.006	.075	.001	.065	.001
Strict		.906	-.002	.905	.001	.075	0	.065	0
Adjusted CU-20									
Configural		.987		.983		.039		.04	
Metric		.987	0	.985	.002	.04	.001	.038	-.002
Scalar		.986	-.001	.984	-.001	.041	.001	.039	.001
Strict		.983	-.003	.981	-.003	.042	.001	.042	.003

Difference of the model from the model before (metric = metric-config; scalar = scalar-metric; strict = strict-scalar)

*CU-40* Chronic Uncertainty Scale-long version, *CU-20* Chronic Uncertainty Scale-short version

**Table 6:** Measurement invariance across different age groups

Model	N	CFI	∆CFI	TLI	∆TLI	SRMR	∆SRMR	RMSEA	∆RMSEA
Age group 1 (18—29)	66								
Age group 2 (30—39)	132								
Age group 3 (40—49)	303								
Age group 4 (50—59)	137								
Age group 5 (60—69)	80								
Age group 6 (70 +)	44								
Adjusted CU-40									
Configural		.846		.832		.081		.09	
Metric		.845	-.001	.839	.007	.097	.016	.088	-.002
Scalar		.832	-.013	.831	-.008	.099	.002	.09	.002
Strict		.826	-.006	.834	.003	.098	-.001	.09	0
Adjusted CU-20									
Configural		.985		.982		.051		.042	
Metric		.986	.001	.983	.001	.06	.009	.04	-.002
Scalar		.980	-.006	.978	-.005	.063	.003	.046	.006
Strict		.966	-.014	.966	-.012	.063	0	.057	.011

Difference of the model from the model before (metric = metric-config; scalar = scalar-metric; strict = strict-scalar)

*CU-40* Chronic Uncertainty Scale-long version, *CU-20* Chronic Uncertainty Scale-short version

#### Short version of the CU

**Fig. 2:**
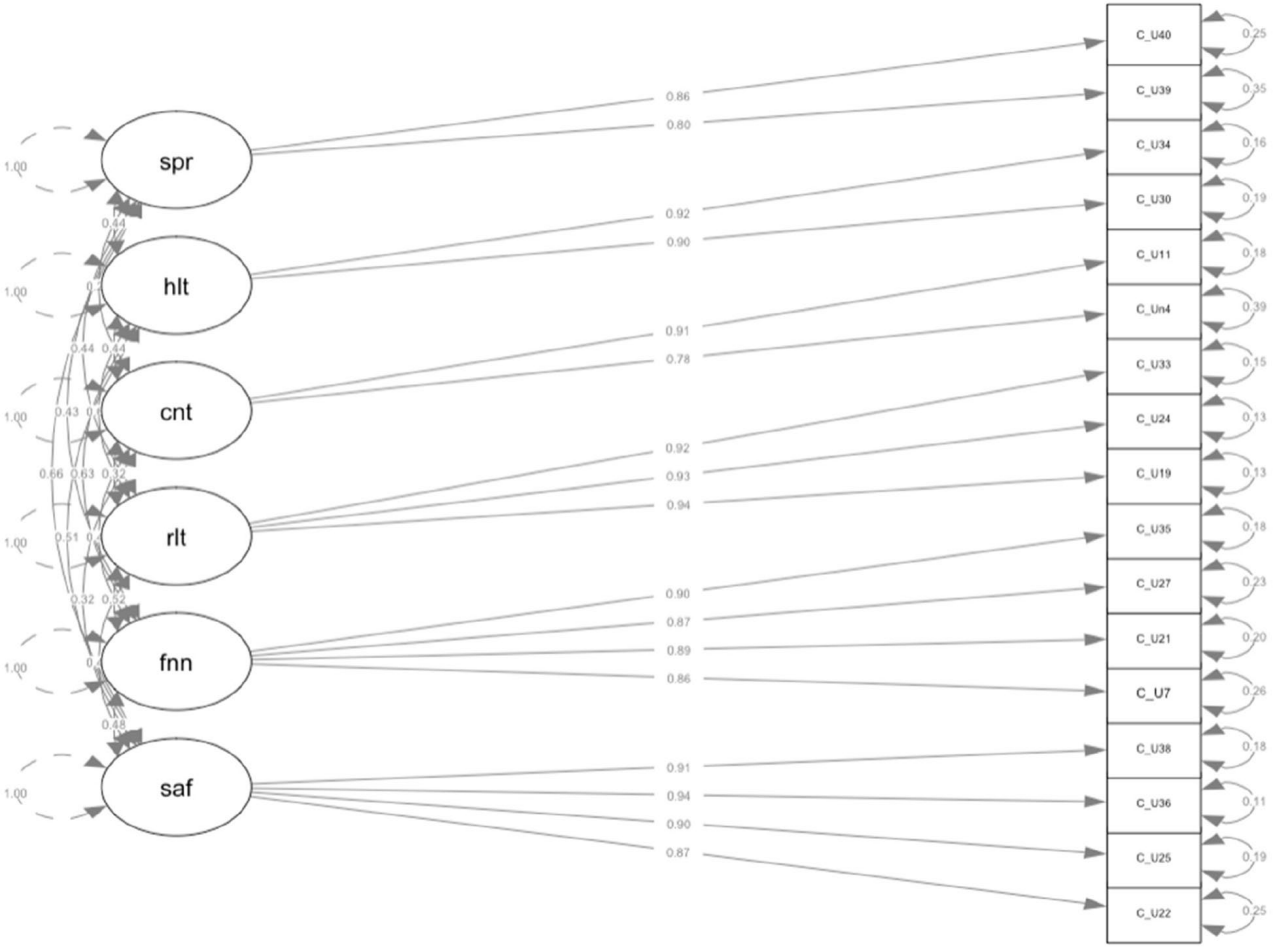
Factor structure of the adjusted Chronic Uncertainty Scale (CU-20) with standardized estimates. Notes. spr = separation from family. hlt = health. cnt = country’s safety and security. rlt = relationship security. fnn = finances. saf = safety

The overall KMO for the CU-20 was 0.91, ranging from 0.82 to 0.94, indicating sample adequacy. Bartlett’s test of sphericity was significant (*X*^2^(19) = 551.22, *p* < 0.001). Using the Principal axis approach, scree-plot and eigenvalue inspection revealed a five-factor structure. Chi-quadrat tests revealed a better model fit for a six-factor structure; hence the six-factor structure was kept. Items were assigned to factors according to the factor loading matrix (Table 7, Fig. 2). Factor estimates ranged between 0.54 and 0.93 (Table 2).

**Table 7:** Exploratory factor analysis: Factor loading matrix – EFA-proposed CU-20

Short version CU	Six-factor structure								
Item	Factor 1	Factor 2	Factor 3	Factor 4	Factor 5	Factor 6	h^2^	u^2^	com
1	.10	.12	-.10	.02	.55	-.08	.36	.64	1.3
4	-.04	-.01	.01	.93	-.03	-.03	.80	.20	1
7	.82	-.06	.03	.06	.05	-.04	.74	.26	1
11	.07	.02	-.04	.76	.01	.05	.65	.35	1
14	.01	.15	-.07	.02	.58	.07	.47	.53	1.2
19	.02	.03	.92	.00	-.01	.01	.87	.13	1
21	.89	.05	-.02	.02	-.02	.00	.81	.19	1
22	.02	.82	-.06	-.03	.03	.09	.77	.23	1
24	-.02	.00	.92	.02	.05	-.01	.87	.13	1
25	.01	.88	.05	-.03	.01	.01	.82	.18	1
27	.87	.02	-.01	-.04	-.03	.08	.78	.22	1
28	.03	.01	.05	.72	.08	.04	.66	.34	1
30	.01	-.02	.04	-.02	.83	.04	.75	.25	1
33	.03	.00	.91	-.01	-.02	.02	.85	.15	1
34	.01	-.02	.06	.04	.83	-.01	.76	.24	1
35	.89	-.01	.03	-.01	.03	-.02	.82	.18	1
36	.03	.96	.03	.03	-.03	-.05	.90	.10	1
38	-.04	.88	.01	.03	.03	.02	.81	.19	1
39	.04	.01	.00	.04	-.05	.82	.69	.31	1
40	-.02	.02	.04	-.02	.08	.80	.72	.28	1

Principal axis extraction, oblimin rotation, h^2^ = communalities of all factors, u^2^ = uniqueness of items, com = Item complexity

*CU-20* Chronic Uncertainty Scale-short version

Model fit of the EFA-proposed six-factor model was good, with CFI = 0.97, TLI = 0.96 SRMR = 0.04, and RMSEA = 0.05 (Table 8). Item 4 (“The country being strong”) and item 28 (“The country’s commitment to protect all of its citizens”) of the subscale *country’s safety and security* correlated highly with the other subscales of the Chronic Uncertainty scale and were hence included as residual covariance. Item 1 (“Being healthy enough to do daily activities”) and item 14 (“Getting through the day without physical struggle”) of the subscale *Health* also correlated highly with the other subscales. Placing them as residual covariance did not lead to further improvements of the model fit. Additionally, item 1 and item 14 had very low standardized estimates (0.54 and 0.61) compared to the other items (standardized estimates > 0.80). Removing item 14 led to better model fit. Additionally, item 22 (“Avoiding harm from violence in your neighborhood”) and item 36 (“Feeling secure in your neighborhood”) correlated highly with other factors and were thus analyzed as residual covariance because they were assigned to the same factor *Safety*. After these adjustments, model fit indices improved: CFI = 0.99, TLI = 0.98, SRMR = 0.04, and RMSEA = 0.05 (Table 8). These adjustments led to better model fit indices than the theoretically proposed 6-factor model (CFI = 0.97, TLI = 0.96, SRMR = 0.04, and RMSEA = 0.05), therefore the adjusted 6-factor model was kept.

**Table 8:** Model fit indices for the short version of the CU-20

	CFI	TLI	SRMR	RMSEA
Theoretical 6-factor model	.97	.96	.04	.05
EFA-proposed 6-factor model	.97	.96	.04	.05
Adjusted 6-factor model	.99	.98	.02	.05

Robust values

*CU-20* Chronic Uncertainty Scale-short version

Measurement invariance analysis revealed metric, scalar and strict invariance across gender: ∆CFI ≤ 0.01, ∆TLI ≤ 0.01, ∆SRMR ≤ 0.03, and ∆RMSEA ≤ 0.015 (Table 5). The short version of the CU questionnaire is metric, and scalar invariant regarding ∆CFI and ∆TLI (≤ 0.01). Values for ∆SRMR ≤ 0.03 and ∆RMSEA ≤ 0.015 indicate metric, scalar and strict invariance (Table 6) across age.

### Reliability

Internal consistency of the CU-40 of the EFA-proposed model and adjusted model was measured with the McDonald’s omega. Both models showed satisfactory to good internal consistencies, with ω ranging from 0.67 to 0.96 (Table 9). When item 20 was removed, McDonald’s omwga remained at 0.963. Across all items, ω when individual items were removed ranged between 0.962 and 0.964.

**Table 9:** Chronic Uncertainty Scale internal consistencies of the EFA-proposed model and adjusted model – Long version CU-40

	McDonald’s omega ω	
	EFA-proposed model	Adjusted model
Safety	.93	.93
Finances	.94	.94
Relational	.96	.96
Country	.87	.88
Health	.82	.81
Mental Health	.67	.67
Total	.87	.87

MBESS

McDonald’s omega for the CU-20 was also good for the EFA-proposed and adjusted model with ω ranging from 0.82 to 0.95 (Table 10). When item 14 was removed, ω remained at 0.932. Across all items, ω when individual items were removed ranged between 0.930 and 0.935.

**Table 10:** Chronic uncertainty Scale internal consistencies of the EFA-proposed model and adjusted model – short version CU-20

	McDonald’s omega ω	
	EFA-proposed model	Adjusted model
Safety	.95	.95
Finances	.93	.93
Relational	.95	.95
Country	.87	.86
Health	.84	.86
Separated	.82	.82
Total	.89	.89

MBESS

## Discussion

Uncertainty is a widespread phenomenon experienced worldwide. If uncertainty cannot be coped effectively, it can become chronic [12] and contribute to mental disorders or other illnesses [41]. Previous research has been primarily focused on transitory or acute forms of uncertainty, often in the context of illness or transitory life episodes, or on dispositional forms of uncertainty. In contrast, chronic experiences of uncertainty and uncertainties that cross multiple targets or dimensions have been mostly overlooked. The current study evaluated the psychometric properties of the short and long version of the Chronic Uncertainty scale (CU-20; CU-40) in a U.S. population.

### Long version CU-40

PCA led to a six-factor solution for the CU-40 with support for the following factors: *1) Safety, 2) Finances, 3) Relationship security, 4) Country’s safety and security, 5) Physical Health, and 6) Mental Health*. Results of this study demonstrate a good model fit for the EFA-proposed CU-40, as well as the adjusted version. Internal consistencies were high for both models. The adjusted model is measurement invariant across both gender and age. In the theoretically proposed CU-40 version, seven factors were extracted, with the additional factors *Other’s well-being* and *Threat of separation from family* [3, 25, 26]. Item 20 (“The people closest to you getting through the day without physical pain”) was originally assigned to the *Other’s well-being* factor, however this item loaded very low to this scale (0.29). According to Tabachnick and Fidell [33], items with factor loadings lower than 30 do not represent a factor, thus item 20 was removed from the current analysis. In the present study, only a six-factor solution was proposed. This might explain why item 20 was loading very low on each of the six factors. Nevertheless, it must be noted that item 20 would fit into the factor *Physical Health* of the adjusted model in terms of its content (see Table 1). Additionally, while the removal of item 20 was based on its low factor loading, the reliability analysis showed that removing this item did not affect the internal consistency of the total scale, with McDonald’s omega remaining high at 0.963. This indicated that item 20 contributed to the overall reliability of the scale but was exclude due to poor factor alignment. Future studies could modify item 20 to analyze if it increases the factor loadings.

Item 5 (“The people closest to you getting through the day without emotional struggle”), item 9 (“The safety and security of the people closest to you”), item 25 (“Your family being safe in your neighborhood”), and item 32 (“The people closest to you having the finances to pay their bills on time”) were also originally assigned to the factor *Other’s well-being*. However, these items were loading higher on the corresponding factors of the adjusted model (see Table 1). Furthermore, item 8 (“Your survival”) and item 9 (“The safety and security of the people closest to you”) from the subscale *Security* were included as residual covariance because the modification indices were loading high on the other factors. After this adjustment, item 9 loaded less with the other factors. However, item 8 still loaded high on the other factors. This might be due to its general phrasing and might need further revision. Moreover, item 18 (“Avoiding being the victim of crime in your neighborhood”) and item 22 (“Avoiding harm from violence in your neighborhood”) from the subscale *Security* were also analyzed as residual covariance because they were loading high on the other factors. This adjustment led to slight improvements of the model fit and both items correlated less with the other factors.

Item 39 (“Your security from arrest) and item 40 (“Your security from being forcibly separated from your family”) were originally related to the factor *Threat of separation from family* of the theoretically proposed seven-factor CU-40 model. Originally this seventh factor was included because the threat of deportation and family separation was salient in the U.S., especially in 2017 and to integrate community-specific uncertainties. However, EFA results subsumed both items under the *Safety* of the EFA-proposed CU-40. Due to the framing of these items, they could fit into the *Safety* factor. EFA might have dropped the seventh factor because there were only two items assigned to it. Future studies could analyze a factor structure with more equally distributed items. Result of simulation studies suggest at least three or four items per factor for better interpretability, more accurate and stable parameter estimates, and more reliable factors [42, 43].

### Short version CU-20

Results of this study demonstrate a good model fit for the EFA- proposed CU-20 and the adjusted model. Internal consistencies were high for both models. The adjusted model is measurement invariant across gender and age. Previous research has yielded similar results [3]. A six-factor solution was also proposed for the CU-20. However, a slightly different item to factor attribution was proposed for the CU-20 compared to the CU-40. The six factors retrieved from PCA were: 1) *Safety*, 2) *Finances*, 3) *Relationship security*, 4) *Country’s safety and security*, 5) *Health*, and 6) *Separation from Family*. According to the EFA, item assignment to factors corresponds to the item to factor distribution of the theoretically proposed model. For the CU-20 the factor *Other’s well-being* was not theoretically proposed because items of the short version did not contribute to the factor.

Item 39 (“Your security from arrest”) and item 40 (“Your security from being forcibly separated from your family”) were attributed to *Separation from Family*. This corresponds with previous research (16) and the theoretical considerations of this factor. It might be that this factor was seen in the CU-20 and not in the CU-40 because of statistical properties tied to the number of items per factor. Moreover, these items were subsumed under *Security* in the CU-40. Nevertheless, the factor retention for the CU-20 in the current paper better differentiates between the two factors *Security* and *Separation from Family*. Given the distinction between uncertainty about security, broadly speaking, and uncertainty about the particular experience of separation, that differentiation is important. Future studies could analyze further modifications to these factors to examine the possibility of improved distinction in the long version of the CU, as well.

Another difference between the short and long version was as follows: In the short version, items measuring mental and physical health both loaded onto the broader *Health* factor, whereas the analyses of the long version CU suggested a distinction between physical and mental health, reflecting two different factors. Broadly, though, the factor structures for the short and long version were mostly similar, and certainty consistent with theoretical intentions of the measures.

Item 14 (“Getting through the day without physical struggle”) was removed from the current study because it correlated highly with the other factors and had relatively low standardized estimates (0.61). Interestingly, item 14 had higher standardized estimated in the long version of the CU (0.85). Despite its removal, item 14 contributed to the overall reliability of the CU-20, as shown by a stable McDonald’s omega of 0.932. However, placing item 14 with item 1 (“Being healthy enough to do daily activities”) as residual covariance did not lead to model fit improvements. Item 14 may not contribute enough incremental validity to the short version of the CU and would thus need a modification. The removal of item 14 was driven by its insufficient fit within the current factor structure rather than its reliability. Future modifications of item 14 or the inclusion of alternative items may enhance the fit of the CU-20 model. It is also essential to consider that item 14 was originally incorporated to measure uncertainty in refugee camps or at natural disasters [25, 26], which may not apply equally or appropriately across populations. It is important to note that the CU was developed with awareness that many targets of uncertainty are universal, but that others are specific to particular community experiences. As such, researchers are encouraged to be aware of unique uncertainty experiences of the community in question and consider adding relevant questions using the same kernel shared by other items in the measure.

Furthermore, item 4 (“The country being strong”) and item 28 (“The country’s commitment to protect all of its citizens”) from factor *Country’s safety and security* had also high covariance factor loadings with the other factors. Given that these items cover other topics like safety, finances, and separation from family, the high covariance to the other subscales is understandable. Item 28, in particular, might be too specific and focused on life-threat, political and social danger. This item stems from the theoretical model that was also intended to measure uncertainty in refugee camps or at natural disasters [25, 26]. Additionally, item 28 has implications on political and social sources of threat, which is relevant for refugees or survivors of natural disasters. The current sample, however, is not comprised of refugees or survivors of natural disasters, which might explain the deviations from the EFA-proposed and adjusted models in this paper from the theoretically proposed CU. Nevertheless, considering recent crises like the COVID-19 pandemic that necessitate governmental support and guidelines, this item is very useful and can thus be adapted to different populations or situations. This corresponds with interpretations of Schmalbach et al. [3].

To the best of our knowledge, this is the first study evaluating the psychometric properties of the CU-20 and CU-40 in an U. S. population. A key strength of the CU scale is its ability to capture prolonged and structural aspects of uncertainty. A high score on the CU scale reflects that individuals have experienced life conditions where their ability to predict important outcomes has been hampered for months or years. This typically results from structural factors that constrain their ability to improve their situation and thereby reduce their uncertainty. Examples include refugees, undocumented immigrants, individuals living under extended conflict, foster children, people living with domestic violence with low income, and those affected by a global pandemic. This construct is not meant to capture transitory or dispositional experiences of uncertainty, but rather indicates a chronic state of uncertainty rooted in long-term structural conditions. The CU framework, which is currently under development by Afifi and colleagues, begins with a critical recognition that structural factors are the primary cause of chronic uncertainties, and should be essential in designing interventions. Such interventions could directly address the structural factors causing chronic uncertainties or improve perceived control and efficacy among affected individuals and communities.

### Limitations

Although one strength of the sample is that it is broader than the typical college sample used within many US-based studies [45], the use of MTurk samples has also come under some critique, with arguments that they are not particularly representative of the U.S. population, at large [44]. Perhaps a related limitation of the study is the non-normality of the data, which may fairly reflect the average individual’s experience with chronic uncertainties, but nevertheless can negatively impact the statistical power of the analysis.

Another important consideration is the conceptual distinction between uncertainty and related constructs like chronic stress. While uncertainty and stress are often correlated, they are empirically distinct. For instance, certainty about negative outcomes (e.g., certainty that one will lose their home in a disaster) can induce stress independently of uncertainty, whereas uncertainty about positive outcomes may generate anticipation or hope rather than stress. These nuances highlight the importance of continuing to refine the CU scale to better capture the specific construct of chronic uncertainty. Additionally, uncertainty can be conceptualized both as a trait and as a state. While the current study focuses on chronic uncertainty as a relatively stable state, further research could explore how this construct interacts with trait measures of uncertainty such as *Intolerance of Uncertainty* [17] in affecting responses. This could provide a more comprehensive understanding of how chronic uncertainty functions in different contexts and over time.

A notable limitation of the current study is the low mean scores of the CU scale items within the general US population, which reflects relatively low levels of chronic uncertainty in this sample. This is expected in a stable and wealthy country like the USA, where many individuals experience less chronic uncertainty compared to more vulnerable populations. The low mean scores might suggest a floor effect, indicating that the scale might not fully capture higher levels of chronic uncertainty experienced by more vulnerable sub-populations, such as lower-income communities, individuals experiencing violence, or refugees. This limitation could potentially underrepresent the strength of the scale and its ability to detect higher levels of chronic uncertainty in these groups. To address this, future research could analyze the factor structure of the CU scale in more diverse or vulnerable sub-samples to validate whether the scale holds across different contexts. Additionally, examining income data and other demographic factors might provide further insights into how chronic uncertainty varies among different populations. Despite these limitations, there is no theoretical reason to believe that the factor structure or the associations among items would be significantly impacted by the mean scores alone. The CU scale is designed to measure chronic uncertainty consistently across different levels of this construct. Therefore, its ability to capture chronic uncertainty should remain robust, even if mean scores are low in the general population.

## Conclusion

The results of this study indicate good model fit and reliability for the EFA-proposed CU-20 and CU-40 and the adjusted models. Further assessments and implementations of the CU-20 and CU-40 in different contexts using a diverse sample is required to examine if the CU can be applied as a multi-dimensional tool to measure simultaneous experiences of multiple chronic uncertainties in a variety of populations.

## Supplementary Information

Supplementary Material 1.

## Data Availability

The datasets used during the current study are available upon reasonable request. Please contact w-afifi@ucsb.edu for further information.

## Abbreviations

Covid-19: Corona virus
CU: Chronic Uncertainty
CU-20: Chronic Uncertainty scale-20-item
CU-40: Chronic Uncertainty scale-40-item
CFA: Confirmatory Factor Analysis
CFI: Comparative Fit Index
EFA: Exploratory Factor Analysis
ML: Maximum likelihood
MGCFA: Multigroup confirmatory factor analysis
MTurk: Amazon Mechanical Turk
SRMR: Standardized Root Mean Square Residual
U.S.: United States
