# Supplementary material for: Psychometric properties of the American version of the Chronic Uncertainty scale: long and short version (CU-40; CU-20)
Source: BMC Psychol. 2024 Nov 27;12:699. doi: 10.1186/s40359-024-02206-6 (PMC11603752; doi:10.1186/s40359-024-02206-6)
Supplement: Supplementary file 1 — Supplementary Material 1. [file 40359_2024_2206_MOESM1_ESM.pdf]

Full version (40 items) – Used in US national sample

The following questions ask about your level of chronic uncertainty about several aspects of your life. Uncertainty is a belief that you're not able to predict the outcome of an issue. When something is chronic, it means that it is ongoing. In each case, you will be asked to think about the average level of uncertainty you have experienced during the past year.

READ CAREFULLY. Please use the following scale in each case. 6 Extremely Uncertain 5 Mostly Uncertain 4 Somewhat Uncertain 3 Somewhat Certain 2 Mostly Certain 1 Extremely Certain

Let's start. Please think about your level of uncertainty about each of the following issues, using the above scale. For those questions that ask you think about "your relationship," please think about your relationship with your dating or marital partner; if you have neither, think of the relationship you would turn to in times of need. **How uncertain have you been in the past year about...**

1. Being healthy enough to do daily activities. [H]
2. Your safety and security from harm. [S]
3. Your relationship being on solid ground. [R]
4. The country being strong. [C]
5. The people closest to you getting through the day without emotional struggle. [O]
6. Your security from being physically attacked. [S]
7. Sustaining an income that meets basic needs. [F]
8. Your survival. [S]
9. The safety and security of the people closest to you. [O]
10. Waking up without physical pain. [H]
11. The country being financially stable. [C]
12. Your security from being demeaned, insulted, or threatened verbally. [S]
13. Your relationship being there for you when you need it. [R]
14. Getting through the day without physical struggle. [H]
15. Avoiding harmful chemicals and fumes in your neighborhood. [S]
16. Having the finances to pay for your transportation. [F]
17. Your protection from injury due to violence. [S]
18. Avoiding being the victim of crime in your neighborhood. [S]
19. Your relationship being fulfilling to you in the future. [R]
20. The people closest to you getting through the day without physical pain. [O]
21. Having the finances to pay for housing. [F]
22. Avoiding harm from violence in your neighborhood. [S]
23. Getting through the day without deep anxiety. [H]
24. Being happy in your relationship. [R]
25. Your family being safe in your neighborhood. [O]
26. Your security from unwanted sexual advances or sexually obscene remarks. [S]
27. Having the finances to feed yourself and your immediate family (if relevant). [F]
28. The country's commitment to protect all of its citizens. [C]
29. Your job security. [F]
30. Getting through the day without deep sadness. [H]
31. Your security from any type of sexual contact that occurs without your explicit consent. [S]

32. The people closest to you having the finances to pay their bills on time. [O]
33. Your relationship lasting. [R]
34. Getting through the day without emotional struggle. [H]
35. Having the finances to pay your bills on time. [F]
36. Feeling secure in your neighborhood. [S]
37. The country being secure from enemies. [C]
38. Your safety in your neighborhood. [S]
39. Your security from arrest. [SEP]
40. Your security from being forcibly separated from your family. [SEP]

Code:

SEP = Threat of separation from family
S = Safety and security
C = Country's safety and security
F = Finances
H = Health
O = Other's well-being
R = Relationship security

Brief Version (20 items) – Use in German Sample

Safety:
22. Avoiding harm from violence in your neighborhood. [S]
25. Your family being safe in your neighborhood. [O]
36. Feeling secure in your neighborhood. [S]
38. Your safety in your neighborhood. [S]

Finances:
7. Sustaining an income that meets basic needs. [F]
21. Having the finances to pay for housing. [F]
27. Having the finances to feed yourself and your immediate family (if relevant). [F]
35. Having the finances to pay your bills on time. [F]

Relational:
19. Your relationship being fulfilling to you in the future. [R]
24. Being happy in your relationship. [R]
33. Your relationship lasting. [R]

Country:
4. The country being strong. [C]
11. The country being financially stable. [C]
28. The country's commitment to protect all of its citizens. [C]

Health:
1. Being healthy enough to do daily activities. [H]
14. Getting through the day without physical struggle. [H]
30. Getting through the day without deep sadness. [H]
34. Getting through the day without emotional struggle. [H]

Separated:
39. Your security from arrest. [SEP]
40. Your security from being forcibly separated from your family. [SEP]
